# Development of nonfibrotic left ventricular hypertrophy in an ANG II‐induced chronic ovine hypertension model

**DOI:** 10.14814/phy2.12897

**Published:** 2016-09-13

**Authors:** Niklas Klatt, Katharina Scherschel, Claudia Schad, Denise Lau, Aline Reitmeier, Pawel Kuklik, Kai Muellerleile, Jin Yamamura, Tanja Zeller, Daniel Steven, Stephan Baldus, Benjamin Schäffer, Christiane Jungen, Christian Eickholt, Katharina Wassilew, Edzard Schwedhelm, Stephan Willems, Christian Meyer

**Affiliations:** ^1^ Department of Cardiology‐Electrophysiology cNEP, cardiac Neuro‐ and Electrophysiology research group University Heart Centre University Hospital Hamburg‐Eppendorf Hamburg Germany; ^2^ DZHK (German Centre for Cardiovascular Research) Partner Site Hamburg/Kiel/Lübeck Germany; ^3^ Department of General and Interventional Cardiology University Heart Centre University Hospital Hamburg‐Eppendorf Hamburg Germany; ^4^ Department of Diagnostic and Interventional Radiology University Hospital Hamburg‐Eppendorf Hamburg Germany; ^5^ Department of Cardiology and Cologne Cardiovascular Research Centre Heart Centre University of Cologne Cologne Germany; ^6^ German Heart Institute Berlin Cardiovascular Pathology Unit Berlin Germany; ^7^ DZHK (German Centre for Cardiovascular Research) Partner Site Berlin Germany; ^8^ Department of Pathology Rigshospitalet University Hospital of Copenhagen Copenhagen Denmark; ^9^ Institute of Experimental and Clinical Pharmacology and Toxicology University Hospital Hamburg‐Eppendorf Hamburg Germany

**Keywords:** Angiotensin II, CMR, hypertension, left ventricular hypertrophy

## Abstract

Hypertension is a major risk factor for many cardiovascular diseases and leads to subsequent concomitant pathologies such as left ventricular hypertrophy (LVH). Translational approaches using large animals get more important as they allow the use of standard clinical procedures in an experimental setting. Therefore, the aim of this study was to establish a minimally invasive ovine hypertension model using chronic angiotensin II (ANG II) treatment and to characterize its effects on cardiac remodeling after 8 weeks. Sheep were implanted with osmotic minipumps filled with either vehicle control (*n* = 7) or ANG II (*n* = 9) for 8 weeks. Mean arterial blood pressure in the ANG II‐treated group increased from 87.4 ± 5.3 to 111.8 ± 6.9 mmHg (*P = *0.00013). Cardiovascular magnetic resonance imaging showed an increase in left ventricular mass from 112 ± 12.6 g to 131 ± 18.7 g after 7 weeks (*P = *0.0017). This was confirmed by postmortem measurement of left ventricular wall thickness which was higher in ANG II‐treated animals compared to the control group (18 ± 4 mm vs. 13 ± 2 mm, respectively, *P = *0.002). However, ANG II‐treated sheep did not reveal any signs of fibrosis or inflammatory infiltrates as defined by picrosirius red and H&E staining on myocardial full thickness paraffin sections of both atria and ventricles. Measurements of plasma high‐sensitivity C‐reactive protein and urinary 8‐iso‐prostaglandin F_2*α*_ were inconspicuous in all animals. Furthermore, multielectrode surface mapping of the heart did not show any differences in epicardial conduction velocity and heterogeneity. These data demonstrate that chronic ANG II treatment using osmotic minipumps presents a reliable, minimally invasive approach to establish hypertension and nonfibrotic LVH in sheep.

## Introduction

Hypertension represents a major public health problem leading to hypertensive heart disease and is associated with a reduction in overall life expectancy (Mozaffarian et al. 2015). The subsequent development of left ventricular hypertrophy (LVH) is a strong risk factor for cardiac arrhythmias and sudden cardiac death independent of hypertension (Wachtell et al. 2007). LVH has long been considered as an adaptive process of the heart compensating for pressure overload in the progression of hypertensive heart disease (Drazner 2011). However, many of its concomitant effects were recently recognized to be related to myocardial fibrosis, where excessive deposition of extracellular collagen results in stiffening of the ventricles and electrical uncoupling, leading to diastolic heart failure and cardiac arrhythmias (Rohr 2009; Creemers and Pinto 2011).

Cardiovascular magnetic resonance (CMR) imaging is currently the accepted reference method for noninvasive quantification of cardiac volumes, mass, and function (Bellenger et al. 2000; Hundley et al. 2010) and is increasingly used in large animal models (Tschabrunn et al. 2015). Sheep are widely employed as animal models for the human cardiovascular system, as they possess an electrical and hemodynamical physiology similar to humans (Meyer et al. 2010; Dardenne et al. 2013; Eickholt et al. 2013). ANG II treatment is well established in small animals (Charles et al. 1995; Stevens and Lumbers 1999), but effects over several weeks regarding cardiac function and remodeling in sheep remain unknown. Therefore, the aim of this study was to establish an ovine hypertension model using chronic ANG II treatment and characterize its effects on cardiac remodeling using CMR, electrophysiological, histological, and molecular biological methods.

## Methods

Experiments in this study were performed with 18 ewes, aged 2–4 years. Animals were handled in accordance with the institutional guidelines and experiments were approved by the regional regulatory authorities. The study conforms to the Guide for the Care and Use of Laboratory Animals, eighth edition, updated by the US National Research Council Committee in 2011.

### Study protocol

**Figure 1:**
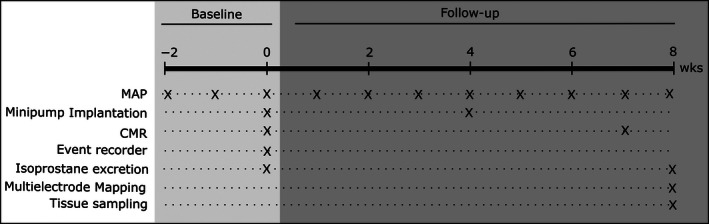
Study protocol. Mean arterial blood pressure (MAP) was measured weekly throughout the experiment, starting 2 weeks before the implantation of osmotic minipumps (baseline). Cardiovascular magnetic resonance was performed in the same procedure as pump implantation and after 7 weeks of treatment (follow‐up). Two animals were implanted with cardiac rhythm monitoring devices. After 8 weeks, all animals were electrophysiologically characterized using epicardial multielectrode mapping.

Figure 1 shows the protocol of the study. Ewes were left to acclimatize for 2 weeks in the animal facility, before being assigned to ANG II (*n* = 9) and control group (*n* = 7) in a randomized fashion. Baseline blood pressure monitoring started 2 weeks before the implantation of osmotic minipumps. Pumps were replaced after 4 weeks. Animals were treated for 8 weeks, in addition, two animals were treated for 16 weeks. CMR was performed directly after pump implantation and 1 week before final open‐chest epicardial multielectrode mapping.

### Animal preparation and anesthesia

Animals were fasted 24 h before all interventions. Anesthesia was initiated with intramuscular injections of 0.5 mg/kg diazepam (B. Braun, Melsungen, Germany) and 7.5 mg/kg ketamine (Albrecht GmbH, Aulendorf, Germany) for intubation and maintained with 1.5% isofluorane (Abbvie, North Chicago, IL) while being ventilated with a mixture of air and 35% oxygen (Julian, Drägerwerk, Lübeck, Germany). During procedures, ECG and oxygen saturation monitoring as well as noninvasive blood pressure measurements (Eagle 4000 monitor, Marquette Hellige, Freiburg, Germany) were performed. At the beginning of the procedure, preoperative analgesics (4 mg/kg carprofen [Zoetis Inc., Florham Park, NJ] and 0.001 mg/kg buprenorphine [Bayer Vital GmbH, Leverkusen, Germany]) were administered intravenously. An antibiotic prophylaxis (2.5 mg/kg enrofloxacin, WDT, Garbsen, Germany) was given for implantation and replacement of minipumps.

For final open‐chest epicardial mapping, anesthesia was induced with intramuscular injection of 0.3 mg/kg midazolam (Roche Pharma AG, Grenzach) and intravenous application of 0.4 mg/kg propofol (B. Braun), followed by continuous delivery of propofol (1–1.5 mg/kg per hour), fentanyl (0.01–0.025 mg/kg per hour, Janssen‐Cilag, Neuss, Germany), and midazolam (1–2.5 mg/kg per hour) by infusion pumps. A 6F vascular sheath (St. Jude, Plymouth, MA) was inserted into the left femoral artery for invasive blood pressure monitoring.

### Implantation of osmotic minipumps

Osmotic minipumps (2ML4, ALZET, Cupertino, CA) were loaded with ANG II acetate (Sigma‐Aldrich, St. Louis, MO) dissolved in 0.9% NaCl according to body weight to achieve a dose of 500 ng/kg per hour (ANG II group) or 0.9% NaCl (control group), respectively (McMahon et al. 1999; Odenbach et al. 2011; Brand et al. 2013; Lemley et al. 2013). Catheters (#0007730, ALZET) were attached to the minipump, followed by overnight equilibration in 0.9% NaCl.

A 4‐cm dermal incision was made at the left side of the throat. After tissue dissection, the catheter was inserted into the left internal jugular vein and closed with a purse‐string suture. The minipump was implanted in a subcutaneous pouch and the incision was sutured with Vicryl 3‐0 (Ethicon Endo‐Surgery, Cincinnati, OH). After 4 weeks, pumps were replaced.

### Implantation of continuous cardiac rhythm monitoring devices

Cardiac rhythm monitoring devices (Reveal LINQ, Medtronic, Minneapolis, MN), were implanted for heart rate measurements and arrhythmia detection in two animals for a duration of 16 weeks (Pürerfellner et al. 2015; Tomson and Passman 2015). Devices were implanted in a subcutaneous pocket on the left lateral thorax by making an incision using the manufacturer's device. The long axis of the device was orientated perpendicular to the ribs. Sensitivity of the device was set to the lowest level. Readout of the devices was performed manually twice per week and additionally with the Medtronic CareLink system (Minneapolis). ECGs of potential arrhythmic events detected by the device were validated by a cardiologist.

### Blood pressure measurements

Measurement of mean arterial blood pressure (MAP) was performed in conscious animals in their familiar environment using a patient monitor with an oscillometric noninvasive blood measurement unit (Eagle 4000 monitor, Marquette Hellige). After being familiarized with the procedure, animals were gently restrained and cuffs positioned on the left thoracic limb between the elbow and the carpal pad (Mühle et al. 2010). Five consecutive measurements were averaged. In case of disturbances or movement, data were discarded. MAP was recorded 2–4 times/week.

### CMR protocol and data analysis

Animals were anesthetized and ventilated as described above. CMR was performed on a 1.5‐Tesla scanner (Achieva, Philips Medical Systems, Best, the Netherlands). A retrospectively gated short‐axis stack was acquired at breath‐hold for the assessment of left ventricular (LV) volumes, mass, and function using a steady‐state free precession (SSFP) sequence. Typical imaging parameters were as follows: reconstructed voxel size 1.48 × 1.48 × 6 mm, echo time = 1.57 msec, time to repetition = 3.13 msec, flip angle = 60°, parallel acquisition technique = SENSE (factor = 2). Endocardial left‐ and right‐ventricular (RV) and epicardial LV contours were manually traced on end‐systolic and end‐diastolic short‐axis cine‐CMR images from base to apex including the papillary muscles for calculating LV volumes, mass, and function (Schulz‐Menger et al. 2013) using commercial software (ViewForum workstation R5.1, Philips Medical Systems, Best, the Netherlands). Analyses were performed in a blinded manner.

### Oxidative stress assessment

For oxidative stress assessment, urinary excretion of 8‐iso‐prostaglandin F_2*α*_ (8‐iso‐PGF_2*α*_) was quantified by gas chromatography–mass spectrometry as described previously (Schwedhelm et al. 2000; Tsikas et al. 2003) at baseline, 4 weeks, and follow‐up. Briefly, urine was extracted via urinary catheters during every intervention directly after initialization of anesthesia and stored with antioxidant at −80°C. Five nanograms of the internal standard 8‐iso‐[3,3′,4,4′‐^2^H_4_]PGF_2*α*_ were added to 5 mL aliquots of urine before extraction. For immunoaffinity chromatographic extraction of 8‐iso‐PGF_2*α*_, 4 mL columns (Cayman Europe, Talin, Estonia) were used. After derivatization, samples were subjected to GC‐MS analysis. Urinary isoprostane excretion rate was expressed in relation to urinary creatinine in order to account for the influence of renal excretory function on urinary isoprostane excretion (Schwedhelm et al. 2004).

### Epicardial multielectrode mapping

Access to the heart was obtained via left lateral thoracotomy and cross‐like incision of the pericardium. A flexible multielectrode array (MEA, EcoflexMEA128, Multi Channel Systems, Reutlingen, Germany: 128 channels, electrode diameter 100 *μ*m, interelectrode distance 2.7 mm, bipolar stimulation site upper left corner, see Fig. 4D) was then consecutively placed on left and right atria and ventricles (LA, RA, LV, RV). Localization on the atria was on the left and right atrial appendages. For the ventricles, electrodes were placed on the anterior side of the heart, about 2 cm above the apex to the septum. After obtaining optimal MEA contact, 2 sec of unipolar electrograms were recorded using a ME128‐FAI‐MPA‐System with MC_Rack software (Multi Channel Systems) in sinus rhythm and during pacing at 120 bpm at each location, from which one pacing train was used for analysis. A sampling rate of 25 kHz was applied; data were digitized with 12 bit. Atrial conduction velocity (CV) was characterized using two parameters: wave propagation velocity (WPV) and conduction heterogeneity index (CHI) (Lau et al. 2010). WPV was quantified calculating conduction vectors within each triangle formed by a triplet of electrodes following the formulation in Kojodjojo et al. (2006). Conduction heterogeneity was assessed using the phase‐mapping method during pacing as described previously (Lammers et al. 1990). The largest difference in activation time with respect to neighboring data points at each site was used to create a phase map and histogram. The CHI was defined as: (P95‐P5)/P50, where P5 is 5th percentile, P95 is 95th percentile, and P50 is median value.

Activation recovery interval (ARI), a surrogate measure for action potential duration, was measured from unipolar electrograms as described in Vaseghi et al. (2013). Briefly, ARI was defined as the time difference between the timing of the maximum positive slope of the repolarization wave and the timing of the maximum negative slope of the intrinsic deflection.

### Postmortem analysis and tissue preparation

Ventricular and septal wall thicknesses were determined 10 mm below the atrioventricular valve plane. For protein and histological analyses, full thickness samples were extracted from left and right atrium, septum, right ventricle, and left ventricular endo‐ and epicardium. Samples were shock frozen in liquid nitrogen for protein extraction and immersed in 10% neutral‐buffered formalin (Sigma‐Aldrich) for histological analyses. After fixing for 4 days at 4°C, tissues were embedded in paraffin.

### Fibrosis quantification

Four‐*μ*m thick tissue sections were cut from paraffin‐embedded biopsies and stained with picrosirius red (de Jong et al. 2012). Fibrosis was quantified using standardized semiautomatic image analysis software. Images of 12 randomly chosen consecutive high‐power fields (×200 magnification), which equal an area of 1 mm^2^, were obtained with a Nikon Eclipse E400 light projection microscope (Nikon, Minato, Tokyo, Japan). Endocardium, areas of fibrous septa radiating from the endocardium into the myocardium, perivascular spaces along intramyocardial arteries, and epicardial fat were avoided. Images, showing artifacts from cutting or staining as well as high‐power fields which contained empty (unstained) spots, were exempt from analysis. Subsequent image analysis was performed to determine the level of interstitial and perivascular fibrosis. This was based on the arrangement of the collagen fibers regardless of staining intensity (dark red shades were indicative of established fibrosis, light red shades were indicative of newly formed fibrosis). The collagen content was further quantified using Nikon software (Nikon Advanced Research NIS Elements imaging software, NIS elements AR 4.10.02, Nikon). The level of interstitial fibrosis, which is defined as collagen depositions between the cardiomyocytes, including perivascular fibrosis, defined as collagen surrounding arterioles, was calculated as the collagen volume fraction (%) per square millimeter (overall fibrosis). Scars, regardless of perivascular or interstitial location, which were defined as double the diameter of a medium‐sized cardiomyocyte in the measured high‐power field, were manually eliminated during the measurement process, using a specific software application tool, in order to obtain purified results for the interstitial fibrosis level. The number of infiltrating leukocytes was eyeballed by an experienced pathologist and graded according to the Dallas criteria on hematoxylin and eosin (H&E)‐stained sections. All histological quantifications were performed blinded.

### Western blot analyses

Tissue biopsies were lysed in RIPA buffer (50 mmol/L Tris base pH 8.0, 150 mmol/L sodium chloride, 0.5% sodium deoxycholate, 0.1% sodium dodecyl sulfate [SDS], 1% Triton‐X‐100, 1 mmol/L dithiothreitol, plus protease inhibitors [Complete Mini, Roche Life Science, Basel, Switzerland] and phosphatase inhibitors [phosSTOP, Roche Life Science]) using an Ultra‐turrax homogenizer (IKA, Staufen, Germany) and centrifuged at 15,000*g* for 30 min at 4°C. Protein concentrations in the homogenates were quantified using the Pierce BCA Protein Assay Kit (Thermo Fisher Scientific, Waltham, MA) according the manufacturer's instructions. Separation of 40 *μ*g total protein was performed under denaturing conditions by sodium dodecyl sulfate–polyacrylamide gel electrophoresis (SDS‐PAGE) using precast gels (4–20%, Bio‐Rad, Herkules, CA) and transferred to nitrocellulose membranes via wet blotting (Laemmli 1970). Membranes were blocked in 5% skim milk powder in Tris‐buffered saline (TBS). Membranes were incubated overnight at 4°C with primary antibodies (anti‐MPO heavy chain 1:500, #sc‐52076, Santa Cruz, Dallas; anti‐collagen I 1:1000, #234167, EMD Millipore Cooperation, Temecula; anti‐TGF‐*β* 1:100 Santa Cruz sc‐146, Abcam plc, Cambridge, UK; phospho‐p38 MAPK 1:1000, cell signaling, #4511; phospho‐p44/42 MAPK [Erk1/2] ERK 1:1000, cell signaling, #4370) in 0.5% milk/TBS. After three washing steps with TBS + 0.5% Tween (TBST), secondary antibody incubation (goat anti‐rabbit POX, PI‐1000, goat anti‐mouse POX, 1:10,000, Vector Laboratories, Burlingame, CA) in 0.5% milk/TBS followed up for 1 h at room temperature. The Fusion Solo S gel documentation system (VWR International, Radnor, PA) was used to detect reactive protein bands with enhanced chemiluminescence.

### Statistical analyses

Variables are reported as mean ± standard deviation. Testing for normality was not performed due to small sample size, instead Gaussian distribution was assumed. Data were therefore compared with Student's *t*‐test. A *P* < 0.05 was considered significant (*≤0.05, **≤0.01, ***≤0.001). Graphpad Prism 5 (Graphpad Inc., La Jolla, CA) was used for statistical analyses.

## Results

### Stable elevation of blood pressure during continuous ANG II treatment

**Figure 2:**
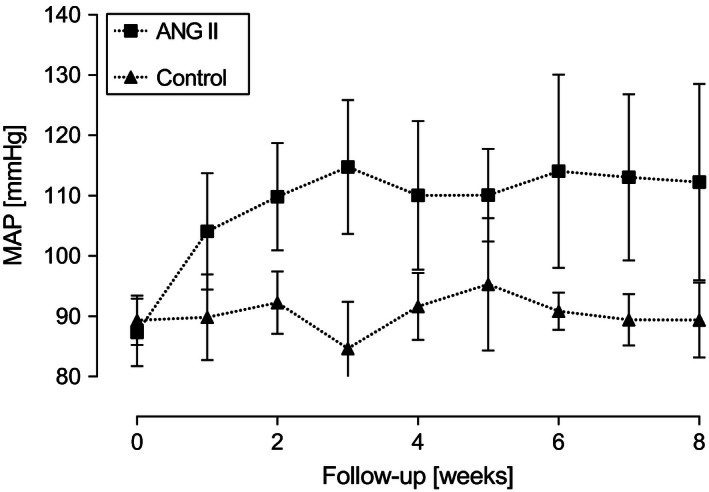
Development of mean arterial blood pressure (MAP) during follow‐up. MAP increases in animals after implantation of ANG II‐containing osmotic minipumps (*n* = 9), whereas no changes were detected in vehicle control (*n* = 7). Data are expressed as mean ± SD.

MAP in the ANG II group increased by 27.9% (87.36 ± 5.3 to 111.84 ± 6.9 mmHg, *P = *0.00013) while no significant change was observed in the control group (89.44 ± 3.72 to 90.49 ± 4.19 mmHg, *P = *0.55). As illustrated in Figure 2, MAP increased within 1 week of ANG II treatment and reached a plateau after 3 weeks of treatment. In both groups, ANG II and control, the total body weight did not change during follow‐up (Table 1).

**Table 1:** Basic parameters at baseline and follow‐up

	Control		8‐week Ang II		16‐week Ang II	
	Baseline	Follow‐up	Baseline	Follow‐up	Baseline	Follow‐up
Weight (kg)	73.5 ± 5.4	75.1 ± 6.2	78.8 ± 4.5	77.8 ± 6.1	100.3 ± 2.8	99.4 ± 4.3
Mean arterial blood pressure (mmHg)	89.4 ± 3.7	90.5 ± 4.2	87.4 ± 5.3	111.8 ± 6.9^a^	106.1 ± 2.2	142.3 ± 5.9
Creatinine (mg/dL)	1.0 ± 0.1	1.1 ± 0.2	0.9 ± 0.1	0.9 ± 0.1	1.0 ± 0.3	1.1 ± 0.2
Heart rate (min^−1^)	–	–	–	–	71.5 ± 6.3	71.6 ± 1.3
CRP (mg/dL)	<0.02	<0.02	<0.02	<0.02	<0.02	<0.02

a *P* = 0.00013 versus Ang II baseline; ^#^ *P* *=* 0.00001 versus control follow‐up; all values are mean ± standard deviation.

### Increase in left ventricular mass during continuous ANG II treatment

**Figure 3:**
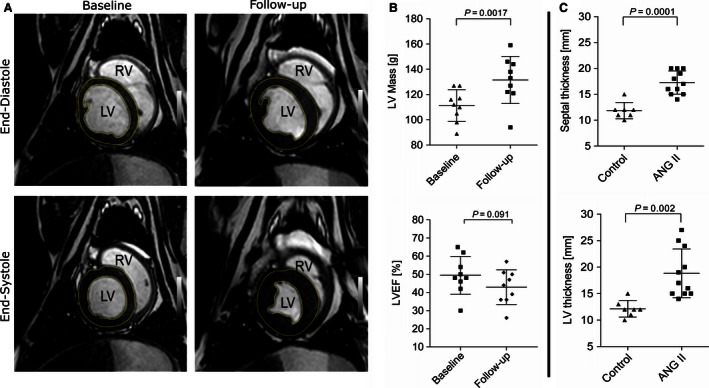
Continuous ANG II treatment leads to development of left ventricular hypertrophy (LVH). (A) An exemplary short‐axis cardiovascular magnetic resonance (CMR) demonstrates the development of LVH from baseline to 7 weeks of ANG II treatment, (B) upper panel: quantification of mean left ventricular (LV) mass via CMR of baseline versus follow‐up shows an increase of 18.3%. Lower panel: a not significant reduction in left ventricular ejection fraction (LVEF) was detectable between baseline and follow‐up. (C) Postmortem measurements of interventricular septum thickness showed an increase of 45.6% in the ANG II group compared to controls (upper panel). Mean LV wall thickness was increased by 55.1% between control and ANG II‐treated group (lower panel). Data are expressed as mean ± SD.

As depicted in Figure 3, LV mass of the ANG II group increased by 18.3% (20.33 ± 13.16 g, *P = *0.0017), whereas no changes were detectable for the control group (−0.2 ± 8.52 g; compare Table 2).

**Table 2:** Functional and anatomical parameters determined by magnetic resonance imaging

	Control		8‐week Ang II	
	Baseline	Follow‐up	Baseline	Follow‐up
Left ventricular mass (g)	109.8 ± 19.9	109.6 ± 18.8	111.2 ± 12.6	131.5 ± 18.7^a^
Left ventricular ejection fraction (%)	45.4 ± 8.8	49.6 ± 11.9	49.4 ± 10.4	42.9 ± 9.5^b^
Stroke volume (mL)	55.6 ± 6.7	51.2 ± 15.51	51.8 ± 12.8	43.8 ± 7.7

a *P* *=* 0.0017 versus baseline.

b *P* *=* 0.092, n.s. versus baseline; all values are mean ± standard deviation.

As presented in Table 2, a statistically not significant reduction in LV stroke volume (51.78 ± 12.76 baseline vs. 43.78 ± 7.71 mL follow‐up, *P = *0.0671) was observed in the ANG II‐treated group, but not in the control group (55.6 ± 6.65 vs. 51.2 ± 15.51, *P = *0.5172). The LV ejection fraction was not significantly reduced in ANG II‐treated animals (49.44 ± 10.43 vs. 42.89 ± 9.54 mL, *P = *0.091; vs. control: 45.4 ± 8.79 to 49.6 ± 11.9 mL, *P = *0.23). Posterior wall thickness measured at 7 weeks in CMR did present a minor, nonsignificant increased wall thickness (9.98 ± 2.02 mm ANG II vs. 9.5 ± 1.11 mm control). Postmortem LV wall thickness was increased in the ANG II group compared to controls (18.82 ± 4.6 mm vs. 12.14 ± 1.57 mm, *P = *0.002). In addition, interventricular septal wall thickness was higher in the ANG II than in the control group (11.86 ± 1.57 mm vs. 17.27 ± 2.24 mm, *P = *0.0001).

### No electrical remodeling during continuous ANG II treatment

**Figure 4:**
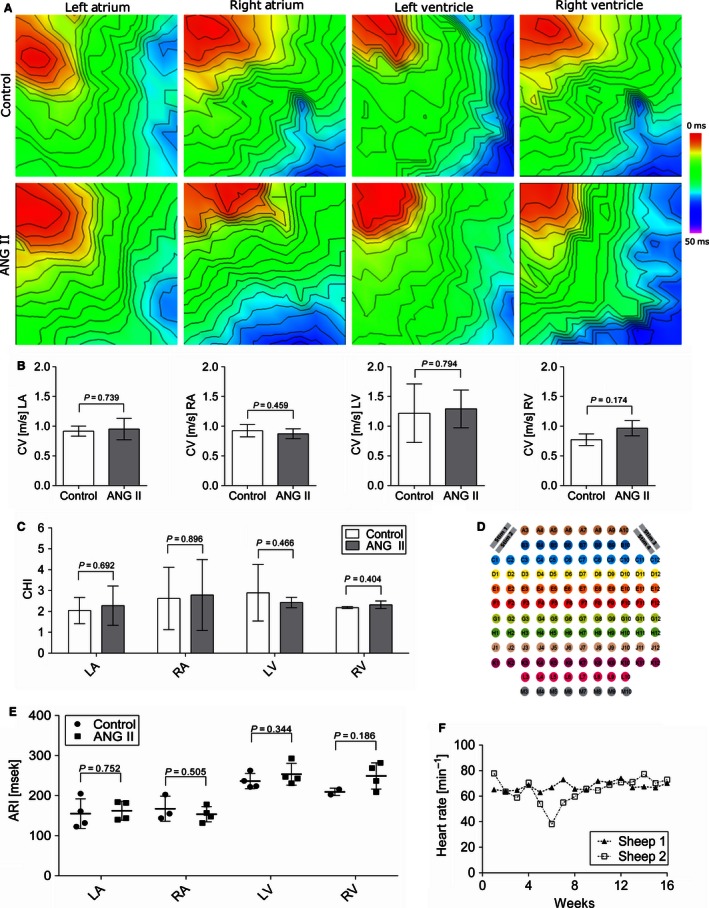
Continuous ANG II treatment does not lead to electrical remodeling after 8 weeks. (A) Representative activation maps from the left and right ventricle and atria with stimulation from multielectrode arrays (MEAs). (B) No change in conduction velocities between ANG II‐treated and control group are detectable (LA *n* = 4 ANG II vs. 4 control; RA *n* = 3 vs. 5; left ventricular *n* = 4 vs. 5, right‐ventricular *n* = 2 vs. 3). (C) Conduction heterogeneity index [(P95‐P5)/P50] of ANG II‐treated versus control group did not indicate any differences. (D) Layout of the flexible MEA with stimulation sites on the upper left and upper right corner. (E) Activation recovery interval of atrial and ventricular myocardium from multielectrode electrograms did not reveal differences between ANG II and control group. (F) Mean daily heart rate of two sheep implanted with cardiac rhythm monitoring devices for 16 weeks of ANG II treatment. A surgical repositioning of the device in sheep 2 after 4 weeks led to an artificial decrease in heart rate between weeks 4 and 8. No change in heart rate was detectable between the onset of treatment and during follow‐up.

Figure 4A shows representative color maps with isochrones depicting points of simultaneous activation. No difference in CV could be detected between ANG II and control group (left atrium 0.92 ± 0.07 vs. 0.95 ± 0.16 m/sec, *P = *0.74; right atrium 0.92 ± 0.08 vs. 0.87 ± 0.07 m/sec, *P = *0.46; left ventricle 1.22 ± 0.43 vs. 1.29 ± 0.0.28 m/sec, *P =* 0.79; right ventricle 0.77 ± 0.07 vs. 0.97 ± 0.1 m/sec, *P = *0.17).

The conduction heterogeneity index was calculated to quantify the scattering of the propagation waves. Despite generally heterogeneous activation patterns, there were no differences between ANG II‐treated and control group in any cardiac region (left atrium 2.04 ± 0.54 vs. 2.28 ± 0.82, *P = *0.69; right atrium 2.62 ± 1.22 vs. 2.79 ± 1.52, *P = *0.90; left ventricle 2.90 ± 1.17 vs. 2.43 ± 0.22, *P = *0.47; right ventricle 2.19 ± 0.04 vs. 2.32 ± 0.15, *P = *0.40). ANG II treatment did not result in conduction changes neither in the left or right ventricle nor in the left or right atrium (Fig. 4B).

For the analysis of atrial and ventricular repolarization, ARI was determined as a surrogate parameter from MEA‐derived electrograms in LA, RA, LV, and RV. As shown in Figure 4E, no differences in myocardial repolarization were found between the groups (ANG II vs. control; LA: 162.5 ± 20.56 vs. 155.25 ± 31.97, RA: 153.75 ± 16.62 vs. 167.33 ± 25.62, LV; 253.5 ± 23.55 vs. 236.5 ± 16.35, RV: 249.25 ± 28.43 vs. 209.5 ± 6.5).

Two animals were treated with ANG II for 16 weeks. In these animals, cardiac monitoring with implanted cardiac rhythm monitoring devices did not show any change in heart rate during follow‐up (71.5 ± 6.3 bpm in week 1 vs. 71.6 ± 1.3 in week 16; Fig. 4F). The observed decrease in heart rate of sheep 2 was attributed to a measurement artifact due to a surgical repositioning of the device after 4 weeks. No arrhythmias were detected during ANG II treatment.

### No fibrotic remodeling and infiltration of leukocytes during continuous ANG II treatment

**Figure 5:**
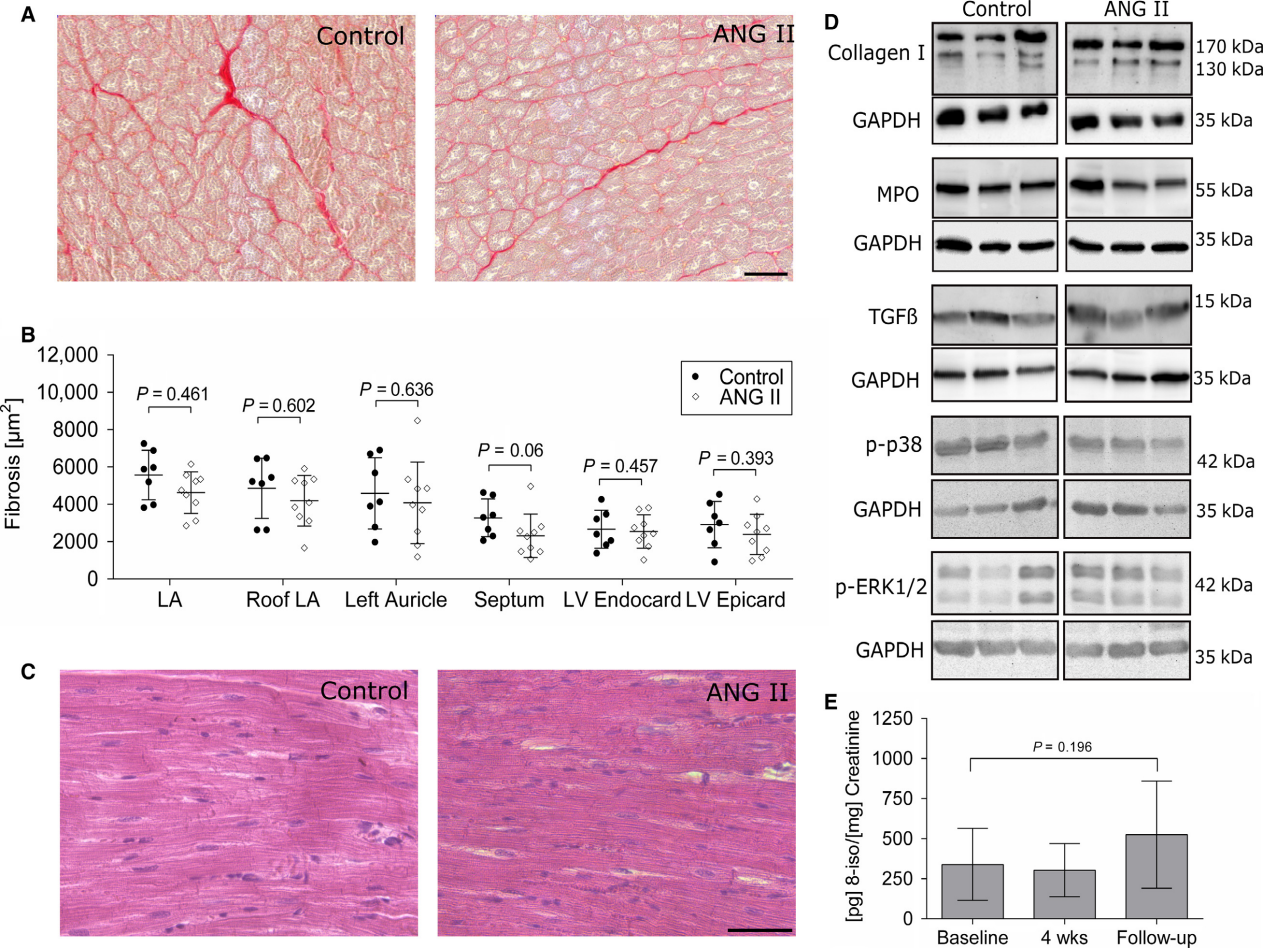
No structural remodeling is detectable after 8 weeks of ANG II treatment. (A) Representative microphotographs of picrosirius red‐stained paraffin sections of left ventricles. Variable amounts of interstitial fibrosis were detectable in the control as well as in the treated group. (B) Amount of cardiac fibrosis based on picrosirius red‐stained tissue sections was quantified using standardized semiautomatic image analysis software. No significant effects between the groups were detectable. (C) Representative H&E stainings of left ventricular samples. ANG II treatment did not lead to an increase in interstitial inflammatory cells. (D) Immunoblotting confirms that neither an accumulation of collagen nor of the leukocyte marker MPO or profibrotic growth factor TGF‐*β* was detectable between the groups. Additionally, no increase in phosphorylation of p38 and ERK was seen in immunoblotting. (E) Urine isoprostane levels were not significantly changed between baseline, 4 weeks, and follow‐up of ANG II‐treated animals indicating no increase in oxidative stress.

Representative examples for picrosirius red‐stained sections are shown in Figure 5A for ANG II and control group. No differences in interstitial fibrosis levels were found in endo‐ and epicardial histological sections of atria, ventricles, and septum between ANG II‐treated and control group (Fig. 5B) and large areas of perivascular scars were not a feature. Figure 5C shows representative H&E stainings of cardiac sections. Regardless of the treatment status, only physiological numbers of scattered mononuclear inflammatory cells were found in a minority of sections indicating no local inflammatory reaction as sign of myocarditis as underlying etiology for fibrous cardiac remodeling. This was confirmed by western blot analysis of ventricular lysates revealing no differences in levels of leukocyte‐derived myeloperoxidase (MPO) between ANG II and control group (Fig. 5D). Fibrous cardiac remodeling is associated with altered expression of collagen type I in particular (Nicoletti and Michel 1999), but we found no differences between ANG II and control group using immunoblotting (Fig. 5D). Furthermore, no elevation of profibrotic growth factor TGF‐*β* was detectable between the groups (Fig. 5D). To check for differences in oxidative stress and fibrosis‐associated signaling pathways, we performed immunoblotting against phosphorylated p38 and ERK1/2. Again, no differences were detectable between the groups (Fig. 5D). High‐sensitivity C‐reactive protein (CRP) levels determined in plasma gave no indication of systemic inflammation in ANG II‐treated animals (Table 1).

As shown in Figure 5E, levels of the oxidative stress marker 8‐iso‐PGF_2*α*_ were not different between baseline, after 4 weeks of ANG II treatment, and follow‐up in the ANG II group.

## Discussion

The major findings of the present study are as follows: (1) Chronic ANG II treatment in sheep results in a stable increase in blood pressure with a subsequent LVH without signs of systemic inflammation. (2) Neither local inflammatory responses nor myocardial fibrosis occurred with a dose of 500 ng/kg per hour. (3) Subsequently, we did not observe electrophysiological remodeling or functional impairment in this ovine hypertension model within 8 weeks.

Hypertension predisposes to cardiovascular morbidity and mortality. Related structural and electrical remodeling of the heart results in an increased risk of ventricular and supraventricular arrhythmias (Peters and Wit 1998; Nishida et al. 2010). Irrespective of hypertension, LVH is a strong risk factor and a hallmark feature for numerous forms of cardiovascular disease (Lazzeroni et al. 2016) and is accompanied by an increased risk for sudden cardiac death (Wachtell et al. 2007). Our presented ovine hypertension model hopefully paves the way to study novel interventional therapies based on the presented minimally invasive approach by using ANG II treatment.

ANG II is well known to not only influence physiological effects such as vasoconstriction and blood pressure regulation, but also to play a significant role in pathophysiological processes such as hypertension and hypertrophy (Mehta and Griendling 2007). In contrast to more invasive hypertension models such as the 1‐kidney–1‐clip model (Lau et al. 2010), ANG II‐based hypertension allows dose adjustments and treatment interruption. Not surprisingly, ANG II treatment has been utilized manifold particularly in rodents (Diz et al. 1983; Nishiyama et al. 2003; Brand et al. 2013), but also in larger animals such as dogs (McKie et al. 2010) and pigs (Matthias et al. 1976). The increase in blood pressure that we observed in our ovine model is comparable to angiotensin infusion in pigs and rats with similar doses regarding the gradual increase as well as the total elevation of blood pressure (Haas et al. 1999; Govender and Nadar 2015). The blood pressure raising effect of ANG II treatment in sheep has already been demonstrated to a similar extent by other groups for up to 1 week, but these studies did not investigate the effect on cardiac function and morphology during a longer follow‐up (Stevens and Lumbers 1999; Hood et al. 2007; Acharya et al. 2011).

In humans, chronic arterial hypertension leads to the development of hypertensive heart disease which is characterized by a marked increase in left ventricular mass and can be associated with local inflammatory response and fibrosis (Weber 2000; Díez 2008). In contrast, other forms of LVH due to intensive exercise, anemia, arteriovenous fistula, or atrial septal defect independent of hypertension are neither accompanied by inflammation nor by fibrosis (Bartosová et al. 1969; Marino et al. 1985; Michel et al. 1986). In an ovine 1‐kidney–1‐clip model, Lau et al. (2010) showed progressive atrial remodeling with structural and functional changes starting with functional impairment and inflammatory infiltrates already from 5 weeks on in the hypertensive, but not the sham‐operated group. While the influence of inflammation on cardiovascular diseases and the induction of arrhythmias in patients is still a matter of debate (Harada et al. 2015), inflammatory processes in murine ANG II‐induced hypertension models were shown to be a prerequisite for fibrosis and arrhythmia induction (Rudolph et al. 2010). Noteworthy, especially the 1‐kidney–1‐clip model is a significant surgical procedure, where a systemic inflammatory response is involved in the induction of hypertension. It can be hypothesized that a systemic inflammation, as it is expected due to the surgical procedure of a nephrectomy, together with hypertension, aggravates the pathology of hypertension alone. This would explain why the model used in Lau et al. did show an early onset of cardiac remodeling and a strong phenotype while having an increase in blood pressure comparable to our model. Meanwhile, implantation of osmotic minipumps is only minimally invasive and we were able to show that it did not result in an elevation of high‐sensitivity CRP and urinary 8‐iso‐prostaglandin F_2*α*_. Surprisingly, despite its role as a profibrotic and proinflammatory factor, ANG II treatment in this study did not lead to inflammatory responses or an increase in phosphorylation of signaling proteins. Consistent with that, TGF‐*β*, an important cytokine in fibrotic remodeling and known to mediate multiple downstream processes, was found unchanged (Rosenkranz 2004; Leask 2010). Whether this would change using a longer treatment remains to be elucidated, but initial results from our studies over 16 weeks of treatment confirmed the previous findings without any signs of inflammation and fibrosis. While studies in ANG II‐treated mice have shown that early inflammatory responses in the form of infiltrating neutrophils releasing S100a8/a9 cause cardiac damage only after 7 days of treatment (Wu et al. 2014), those studies used more than 150× ANG II than our model. A dose‐dependent effect can therefore not be excluded. Still, our study did not analyze different time points during the experiment, only isoprostane levels were analyzed after 4 weeks. It could be speculated that initial inflammatory responses occur very early after the onset of ANG II delivery which are later counterbalanced due to the low dose of ANG II (Zhou et al. 2012).

Whether and to which extent the lack of fibrotic remodeling and related electrophysiological changes in our study can be explained by the lack of systemic inflammatory responses cannot be answered by our data. However, it does confirm other studies by highlighting the importance of inflammation in hypertension and remodeling. Our findings suggest that the hypertensive effect of ANG II is of greater importance for the development of LVH than its proinflammatory effect. For future analyses, it would also be interesting to assess other diagnostic clinical parameters such as members of the natriuretic peptide family. Atrial and brain natriuretic peptides have been shown to be increased in an ovine LVH model of suprarenal aortic banding (Charles et al. 1996). We did not check for atrial hypertrophy, since the spatial resolution of CMR of about 1.5 × 1.5 × 8 mm^3^ is not sufficient to draw meaningful conclusions. It would, however, be interesting to check in future experiments.

Clinically, inhibition of the renin–angiotensin system is first‐line medication in arterial hypertension and has been shown to be superior to calcium antagonists and beta‐blockers with respect to the development of LVH (Brilla et al. 2000; Devereux 2004). Nevertheless, the link between the renin‐angiotensin system and the development of LVH is currently not fully understood. Whether LVH in our model is related to a direct effect of ANG II on the myocardium or to the raise in blood pressure itself cannot be answered by our data, because a group with antihypertensive treatment not targeting the renin‐angiotensin system was not employed in this study. End‐diastolic posterior wall thickness measured via CMR at 7 weeks of treatment is not significantly different between the groups, whereas the difference measured postmortem at 8 weeks is considerable. This is consistent with prenatal dexamethasone‐induced hypertension and LVH in sheep, where an increase in LV mass measured via echocardiography was shown to increase, while wall thickness presented only a minor increase (Dodic et al. 2001). It has to be taken into account that 2D‐based and M‐Mode measurements (where LV mass is calculated based on LV and septal thickness) were shown to lead to a consistent underestimation, thus postmortem measurements are seen as gold standard for wall thickness measurements, while CMR‐based 3D measurements of the whole ventricle were shown to be the best in vivo method for LV mass calculation (Myerson et al. 2002).

## Conclusion

Taken together, we established a reliable model for arterial hypertension in sheep using chronic ANG II treatment via osmotic minipumps over 8 weeks. We demonstrated that this leads to a significant increase in blood pressure and subsequent LVH but no significant alterations in myocardial function. While the model at hand is not suitable to study the effects of hypertension‐induced fibrotic remodeling, it might present a useful tool to further investigate nonfibrotic LVH.

## Conflict of Interest

None declared.
